# Aptamer-Based Screens of Human Body Fluids for Biomarkers

**DOI:** 10.3390/microarrays4030424

**Published:** 2015-09-22

**Authors:** Dania Albaba, Sanam Soomro, Chandra Mohan

**Affiliations:** Department of Biomedical Engineering, University of Houston, 3605 Cullen Blvd, Houston, TX 77204, USA; E-Mails: dalbaba@uh.edu (D.A.); ssoomro2@uh.edu (S.S.)

**Keywords:** biomarker, aptamer, diagnostics, immunoassay, microarray

## Abstract

In recent years, aptamers have come to replace antibodies in high throughput multiplexed experiments. The aptamer-based biomarker screening technology, which kicked off in 2010, is capable of interrogating thousands of proteins in a very small sample volume. With this new technology, researchers hope to find clinically appropriate biomarkers for a myriad of illnesses by screening human body fluids. In this work, we have reviewed a total of eight studies utilizing aptamer-based biomarker screens of human body fluids, and have highlighted novel protein biomarkers discovered.

## 1. Introduction

Antibodies have contributed tremendously to the field of proteomics, especially in applications based on molecular recognition. However, they have shown limitations including the need to first identify specific antibodies to given targets and the possible triggering of immunogenic responses when used as therapeutics. Additional shortcomings include their inability to withstand temperature and environmental changes, the laborious process of their production, and their short shelf life. Further, although antibody-based assays such as sandwich based ELISAs are highly specific for single analyte detection, it has become apparent that there is little possibility of them being used for multiplexed protein detection due to cross reactivity of secondary antibodies [1]. Large-scale antibody based biomarker screens have been done with the use of up to 1000 printed antibodies that bind to biotinylated target proteins in the sample. To date, the largest published study using this platform consists of a microarray capable of screening for 507 proteins, as reported in a couple of studies [2,3]. The challenge in using such an antibody based biomarker detection platform lies in the difficulty in developing single antibodies that are specific and sensitive to the target proteins, with minimal cross-reactivity with the hundreds of other antigen/antibody interactions occurring on the platform concurrently. The development of aptamers and aptamer-based screening assays has resolved some of these shortcomings and has allowed for the expansion of multiplexed targeted proteomic screens.

Aptamers are short single-stranded nucleic acid oligomers (ssDNA or RNA) that bind with high affinity to specific molecular targets [4]. The specificity and characteristics of aptamers are determined by their complex tertiary structure, unlike genetic materials whose primary sequence plays this role. Aptamers are capable of detecting differences in chiral molecules, enabling them to distinguish similar molecules from one another. Further, aptamers are more stable and more resistant to organic solvents than antibodies [5]. Aptamers are selected using Systematic Evolution of Ligands by Exponential Enrichment (SELEX). Using this technique, aptamers with high affinity and specificity to the target can be isolated from the sequence pool after a few rounds of selection.

This review compiles all aptamer-based biomarker screens of human body fluids published to date. These studies are of particular importance because viable biomarkers which can be measured as indicators of pathogenesis are needed for all chronic diseases ranging from Alzheimer’s disease to lung cancer [6]. With the aid of highly multiplexed aptamer-based targeted screens, researchers aim to identify novel biomarkers for non-invasive diagnostics and to gain insights on the pathogenic mechanisms underlying various diseases.

## 2. Technology

### 2.1. Mass Spectrometry

Aptamers have primarily been used in large-scale mass spectrometry (MS) studies to reduce background noise [7]. However, further studies with this technology are needed in order to validate its capability in detecting potential biomarkers. Although mass spectrometry (MS) has been the method of choice for unbiased global proteomic analysis, some of its drawbacks include the overshadowing of low-abundance markers by high-abundance proteins, the low yield with which this technology has uncovered clinically useful biomarkers, and its modest translational potential to routine clinical testing or point-of-care monitoring.

### 2.2. Aptamer-Based Screens Using “SOMAmers”

Almost all multiplexed aptamer-based proteomic screens to date have been executed using the same commercially available platform [1,8]. It utilizes specially modified aptamers, known as SOMAmers (Slow Off-rate Modified Aptamers) for multiplexed protein analysis, called the SOMAscan. SOMAmer reagents are modified short DNA sequences that are analogous to antibodies. They contain functional side-chains that interact and bind to conformational epitopes, specific proteins as well as a 5′-linker for compatibility with the screening platform. Because of their slow off-rate modification, SOMAmers can be used with more sensitivity when compared to antibodies. SOMAmers are identified using the SELEX process without the use of animals and with more cost-effective means in contrast to the generation of antibodies. Contrary to antibodies, aptamers, including SOMAmers, are able to withstand extremes of temperature and pH, and they can easily be desiccated, rehydrated, denatured, and re-natured without any activity loss [8].

In a typical multiplexed screening application, three serial dilutions of the sample are probed with SOMAmer mixtures to assay high, mid, and low abundant proteins. This uniquely allows for the technology to cover a large dynamic range, detecting proteins from μM to fM concentrations. After SOMAmer to target binding in the sample, a series of washes remove unbound SOMAmers and a competitor solution takes advantage of the slow off rate binding of SOMAmers to remove any nonspecific interactions that would have higher kinetic rate constants. The remainder of the assay involves a combination of the three reaction mixtures resulting in the detection of bound aptamers on a DNA microarray printed with anti-SOMAmer probes. The usage of a printed DNA microarray and individual SOMAmers allow for the creation of custom SOMApanels which can target specific protein pathways or classes.

Compared to bead-based immunoassays, which have sensitivities of 1–1000 pg/mL, plate-based sandwich immunoassays, which have sensitivities of 100 pg/mL, and MS-based monitoring with enrichment, which has a sensitivity of greater than 1000 pg/mL, the commercially available aptamer-based proteomic screen is able to detect proteins in plasma with a sensitivity of only about 0.8 to 1.5 pg/mL [8]. With regards to the number of proteins that can be interrogated in each assay, the commercial aptamer-based screen has the capacity to interrogate >1000 proteins, thus exceeding MS-based MRM with enrichment (75–150 proteins), plate-based sandwich immunoassay (100 proteins), printed antibody arrays (1000 proteins), and bead-based sandwich immunoassay (262 proteins) protocols.

The number of proteins that can be interrogated in parallel using aptamer cocktails is also the current bottleneck in the technology. Although currently ~1000 aptamers can be used in a cocktail to assay the level of ~1000 corresponding protein targets without being compromised by cross-reactivities or sensitivity and specificity issues, whether this number can be scaled up to eventually encompass the entire proteome is an open question. This entails not only the generation or selection of aptamer ligands for each protein in the proteome but also the careful validation of its use in multiplexed cocktails.

Regardless, the current technology, as it is, offers the most comprehensive coverage in targeted proteomics and has already yielded interesting insights, as reviewed below. Additional promise of this technology is seen with the commercial availability of over 250 SOMAmer reagents to date for use in immunohistochemistry, flow cytometry, ELISA, and affinity purification allowing for further translation of biomarker findings using the SOMAscan assay.

## 3. Aptamer-Based Biomarker Discovery Studies

### 3.1. Neurological Diseases

Alzheimer’s disease (AD) is a neurodegenerative disease that is progressive and irreversible. As of yet, there is no cure for this illness although there are drugs to help alleviate the symptoms. Two studies utilizing plasma samples have been conducted on Alzheimer's patients, both of which have found clusterin and pancreatic prohormone to be a potential biomarker for AD [9,10]. Clusterin is a glycoprotein with known involvement in Alzheimer’s disease that plays a role in preventing inflammation and reducing apoptosis [11]. Sattlecker *et al.* [9] found that in patients with fast declining Alzheimer’s, clusterin was elevated. Pancreatic prohormone, which has been shown in another study to have specificity for Alzheimer’s, is a regulator of pancreatic and gastrointestinal processes [12]. Other potential biomarkers identified in Sattlecker *et al.*’s study include fetuin B, which is neuroprotective in Alzheimer’s, and PSA-ACT, which has also been investigated for its biomarker potential in Alzheimer’s disease by others [13,14]. Sattlecker *et al.*’s research reported an area under the curve (AUC) of 0.70, with 67% sensitivity and 64% specificity for a panel of 13 proteins. In Kiddle *et al.*’s research, the most predictive set was that of covariates with the most overrepresented proteins (as listed in Table 1), which showed that the biomarker set has a sensitivity of 80% and specificity of 72% [10]. Further validation using longitudinal data sets is warranted for all biomarkers reported in both of these studies to assess their true diagnostic and prognostic significance in Alzheimer’s disease. These studies are summarized in Table 1.

### 3.2. Pulmonary Diseases

Tuberculosis (TB) is a bacterial disease caused by *Mycobacterium tuberculosis* that affects the lungs. Two studies have examined blood samples from patients with tuberculosis, comparing their response to treatment, over time. De Groote *et al.*’s study involved 39 TB patients from Uganda, and showed an increase in proteins that mediate antimicrobial defense and tissue remodeling, with specificity and sensitivity over 90%; the proteins are listed in Table 1 [15]. Another study by Nahid *et al.* [16] found that proteins that were involved in innate and adaptive immunity were differentially expressed in TB patients with specificity of 95% and sensitivity of 90%. A 5-protein classifier was created through the results of their discovery study in order to predict treatment response (as listed in Table 1) with an AUC of 0.8. More studies need to be done in order to determine which set of biomarkers can effectively be used clinically in this disease.

### 3.3. Musculoskeletal Diseases

Duchene muscular dystrophy (DMD) is an X-linked recessive illness that affects 1 in 3600 males. To date, only one study by Hathout *et al.*, has been conducted to mine potential biomarkers for DMD utilizing two cohorts, as is listed in Table 1 [17]. The first compared 42 DMD serum samples and 28 controls; the second compared 51 DMD samples and 17 controls. They found an elevation of three serum proteins, TNNI2, MB, and HSPA1A, and a decrease in RET, GSN and IBSP in the DMD samples in comparison to the controls. An important finding of this study is that these proteins seem to be most significantly altered at a younger age, which suggests that muscle death in DMD patients is highest in the earlier years and is concurrent with the high death rate of DMD patients before the age 25. A limitation of this study includes the lack of any verification or validation of the results; hence, further validation would be required to confirm the results of this study.

### 3.4. Malignancies

Aptamer-based biomarker discovery studies have also been applied to cancer research, from cancers of the pulmonary system to melanoma. In Ostroff *et al.*’s multicenter aptamer-based study on lung cancer in 291 patients within 6 weeks of biopsy or pre-biopsy and 1035 control patients, twelve serum biomarkers were identified as potential classifier proteins with 91% sensitivity and 84% specificity [18]. Since 1326 sera were analyzed, this study constitutes the largest study ever to be conducted using a multiplexed aptamer library for screening! The study was further validated by ELISA assays, which showed 89% sensitivity and 83% specificity for the proteins mentioned in Table 1 [19]. In Mehan *et al.*’s study on serum samples, 94 non-small cell lung cancer (NSCLC) cases and 241 controls were run on Somalogic’s platform [20]. Seven classifier proteins were identified with an AUC of 0.85 for all cases and an AUC of 0.93 for squamous cell carcinoma. The study was then validated blindly using the same aptamer-based technology. The results revealed up-regulation of pathways involved in positive regulation of cell proliferation, breakdown of the extracellular matrix, inflammatory response, and metabolic homeostasis, as well as a down-regulation in amino acid, one-carbon and nitrogen metabolism. Although both the above studies focused on serum from lung cancer patients, different sets of discriminatory markers were identified. This could potentially relate to differences in the histological type of lung cancer studied, the nature of the controls used (smokers *vs.* benign nodules), and ethnic or genetic differences in the study populations. Clearly, both of the studies warrant further validation in additional sample sets using orthogonal platforms.

**Table 1:** Aptamer-Based Biomarker Studies by Body System.

Disease	Subjects ^1^	Body Fluid	Proteins Identified ^2^	Ref.
Neurological Disease	AD (319)	Plasma	A1AT, CCL18, clusterin, (C3), C6, GCSF, (IGFBP-2), ITIH4, (pancreatic prohormone)	[10]
	MCI (149)			
	Controls (209)			
	AD (300)	Plasma	clusterin, (fetuin B), pancreatic prohormone, PSA-ACT	[9]
	MCI (149)			
	Controls (211)			
Respiratory Diseases	Pulmonary TB (39)	Serum	antithrombin III, (CRP), (LBP), (LEAP-1), MRC-2, (NPS-PLA2), (SAA), SEPR, TIMP-2, TSP4	[15]
	Pulmonary TB (39):	Serum	coagulation factor V, ECM1, gp130, TIMP2, XPNPEP1	[16]
	Responders (19)			
	Slow Responders (20)			
Musculoskeletal Diseases	DMD (42)	Serum	(GSN), HSPA1A, (IBSP), MB, (RET), TNNI2	[17]
	Controls (28)			
Malignancies	Lung Cancer (291)	Serum	(cadherin-1), CD30 ligand, endostatin, HSP 90α, (LRIG3), MIP-4, pleiotrophin, PRKCI, (RGM-C), (SCF sR), (sL-Selectin), YES	[18]
	Smoker Controls (1035)			
	NSC Lung Cancer (94)	Serum	C9, (CA6), (CNDP1), CRP, MMP-7, MMP-12, SERPINA-3	[20]
	Smoker/benign Controls (269)			
	Mesothelioma (177)	Serum	(APOA1), C9, CCL-23, CDK-5, F9, FCN2, (FN1), ICAM-2, (KIT), MDK, (SERPINA-4), TNFRSF-8	[21]
	Controls (142)			

^1^ Indicated in parenthesis are the numbers of subjects studied. Abbreviations used: AD, Alzheimer’s Disease; MCI, mid cognitive impairment; TB, tuberculosis; DMD, Duchene Muscular Dystrophy; NSC, Non-Single Cell. All listed aptamer-based screens were conducted using the SOMAscan platform. ^2^ Proteins that are elevated in the diseased samples are not parenthesized. Proteins that were reduced in disease samples are in parenthesis.

Mesothelioma (MM) is an aggressive pleural lining cancer associated with asbestos exposure. In Ostroff *et al.*’s study of 177 mesothelioma serum samples and 142 controls, 13 classifier proteins were identified with a combined sensitivity of 93.2% and specificity of 90.8% [21]. Many of the proteins are involved in inflammation, immune response, cellular adhesion, and proliferation. Four of the proteins, APOA1, FIN1, KIT, and SERPINA4 were found to be down-regulated while the rest as listed in Table 1 were found up-regulated in MM patients in comparison to the controls. A validation study was then used to confirm the results of their discovery, again using the same aptamer-based proteomic technology.

### 3.5. Aging

A couple of studies have utilized aptamers in multiplexed proteomic studies to uncover biomarkers for aging. Through their aptamer-based screens of cerebrospinal fluid (CSF) from 90 adults aged 21 to 85 years old, Baird *et al.* [22] found that among the elevated proteins in aged subjects, most corresponded to inflammation and injury. One such biomarker, the von Willebrand Factor, had the largest increase in relative frequency units (RFU) in the SOMAscan. This finding may be particularly significant because it is a large glycoprotein that is released in response to tissue injury, suggesting increased tissue injury in the nervous system with aging. In another study by Menni *et al.* [23], plasma samples of 206 female twins were analyzed using aptamer-based biomarker technology. This was followed by a replication cohort involving 677 males and females that were diagnosed with ADD, Alzheimer's, or dementia. Menni *et al.*’s research showed two proteins most strongly associated with age: chordin-like protein 1, which is involved with bone morphogenic protein signaling, and pleiotrophin, a growth factor [23]. Of all the proteins interrogated by Menni *et al.* [23], only three showed coordinate changes in the expression of the corresponding genes. Both of these studies need further validation in independent sample sets using orthogonal assay platforms.

## 4. Conclusions

Compared to genomic, transcriptomic, and other proteomic platforms, aptamer-based targeted proteomic screens are still in its infancy, with only eight published studies to date, focusing on human body fluids. Though these limited studies have already unearthed potential biomarkers or biomarker panels, several hurdles remain. Several of the identified protein markers need to be independently validated in additional patient cohorts, using orthogonal assay methods. Second, all of the studies listed in this review were conducted using the same commercial platform utilizing the same library of ~1000 aptamers. Further, all studies were either conducted by Somalogic’s staff or funded by the company. Independently generated aptamer libraries are warranted to corroborate and amplify the findings obtained using the current commercial platform. Third, the comprehensiveness of coverage needs to be expanded beyond ~1000 proteins so that larger fractions of the functional human proteome can be interrogated. Finally, the emerging biomarker screening technologies need to be applied to biological samples procured from prospective longitudinal studies so that prognostic disease markers with clinical utility can be identified. Despite these limitations, aptamer-based targeted proteomics emerge as one of the most promising tools for identifying protein markers with potential clinical significance.
